# Behavioral Interventions Targeting Base of Tongue to Posterior Pharyngeal Wall Approximation: A Scoping Review

**DOI:** 10.1007/s00455-022-10519-0

**Published:** 2022-09-26

**Authors:** Sebastian H. Doeltgen, Rebecca Francis, Stephanie K. Daniels, Harsharan Kaur, Leila Mohammadi, Joanne Murray

**Affiliations:** 1 Speech Pathology, College of Nursing and Health Sciences, Flinders University, Adelaide, Australia; 2 Swallowing Neurorehabilitation Research Laboratory, Caring Futures Institute, Flinders University, Adelaide, Australia; 3 Communication Sciences and Disorders, University of Houston, Houston, TX USA; 4 The Library, Flinders University, Adelaide, Australia; 5 Clinical Library, Campbelltown Hospital, South Western Sydney Local Health District, Sydney, Australia

**Keywords:** Base of tongue, Posterior pharyngeal wall, Dysphagia, Swallowing, Rehabilitation, Exercise, Deglutition, Deglutition disorders

## Abstract

Pharyngeal pressure generated by approximation of the base of tongue to the posterior pharyngeal wall (BOT-PPW approximation) is critical for efficient pharyngeal bolus passage and is a frequent goal of dysphagia management. This scoping review evaluated behavioral interventions available to improve BOT-PPW approximation. We searched MEDLINE, CINAHL, Ovid Emcare, Web of Science, SCOPUS, and ProQuest for studies that met the following criteria: (i) behavioral interventions targeting BOT-PPW approximation, which (ii) were assessed using BOT-PPW-specific outcome measures, and (iiia) performed over a period of time (Review Part 1) or (iiib) studied immediate effects (Review Part 2). Study quality was rated using the GRADE framework. Data were extracted and synthesized into dominant themes. Of the 150 studies originally identified, three examined long-term effects (two single cases studies of individuals with dysphagia, and a third study evaluating effortful swallowing in healthy individuals). BOT-PPW approximation only increased in the two single case studies. Twenty-one studies evaluating immediate effects were categorized as follows: (1) effortful swallowing, (2) Mendelsohn maneuver, (3) tongue-hold maneuver, (4) super supraglottic swallowing maneuver, and (5) non-swallowing exercises. Across all studies, varying levels of success in increasing BOT-PPW approximation were reported. Four of 21 immediate effects studies evaluated patients with demonstrated swallowing impairment, whereas 17 studies evaluated healthy adults. Quality assessment revealed low strength of the existing evidence base. The evidence base for rehabilitative interventions targeting BOT-PPW approximation is severely limited and translation is hindered by small sample sizes and methodological limitations. Further clinical research is warranted.

## Introduction

Impaired swallowing (dysphagia) is a common consequence of many health conditions, including stroke [1], neurodegenerative disease [2], and head and neck cancer (HNC) [3]. Dysphagia negatively affects patient health, safety and quality of life, and increases carer burden [4] and health care expenditure [5].

A common biomechanical characteristic of dysphagia is impaired approximation of the base of tongue (BOT) and the posterior pharyngeal wall (PPW) in the upper pharynx [6]. While impairment in BOT-PPW contact has frequently been detailed in the HNC population [6–8], recent research has identified it as a frequent biomechanical impairment in a large heterogeneous group of patients [9]. Physiologically, BOT-PPW approximation generates the positive driving force that propels the bolus through the pharynx during the pharyngeal phase of swallowing [10–12]. To achieve this contact, contraction of styloglossus, hyoglossus, and palatoglossus muscles is required for retraction of the tongue, while contraction of the superior pharyngeal constrictor muscle pulls the PPW forward. Impaired BOT-PPW approximation commonly results in incomplete pharyngeal bolus clearance, yielding valleculae residue and the potential for post-swallow airway invasion [6].

Of note, even though the sequence of muscle contraction in the pharyngeal phase of swallowing is governed by central pattern generators in the brainstem [13], BOT-PPW approximation, and consequently pressure generation in the upper pharynx, can be modified using volitional maneuvers, including the effortful [14] and Mendelsohn maneuvers [15]. This highlights the potential for targeting impairment of upper pharyngeal pressure generation through behavioral exercises that focus on BOT-PPW approximation.

While some previous reviews have focused on the biomechanical or functional effects of a specific, single intervention (for example [16]), to date no reviews are available that collate and appraise the evidence base across all interventions that aim to improve BOT-PPW approximation. Such a review would be of clinical value in that it would provide a comprehensive resource for clinicians supporting clients with impaired BOT-PPW approximation, while also providing guidance for future research in this area. The aim of this scoping review was, therefore, to explore the following research question: Which rehabilitation interventions are available to improve BOT-PPW approximation during deglutition?

## Methods

We adopted a scoping review methodology to evaluate the existing evidence base for interventions targeting BOT-PPW approximation and mapped the key characteristics of these interventions [17].

### Search Strategies and Inclusion Criteria

We designed the original search in Medline, tested the search with a few key articles, finalized the search, and performed it in Ovid Medline. The master search was then translated into five more databases, CINAHL, Ovid Encare, Web of Science, Scopus, and Proquest (Health and Medicine Collection). The search was undertaken on 17th June 2021, with an updated search carried out on 02nd February 2022 to ensure inclusion of the most recent relevant publications. No limitations were imposed on publication date, and only studies reporting original data, published in English, were included.

The search terms included swallowing disorders, behavioral interventions, rehabilitation exercises, and terms relating to base of tongue to posterior to pharyngeal wall contact (Table 1). We excluded terms relating to surgeries or medical procedures, medication, and animals.

**Table 1:** List of search terms. Search terms used across MEDLINE, CINAHL, Ovid Emcare, Web of Science, SCOPUS, and ProQuest (Health and Medicine Collection)

Search terms used across the databases		
Relevant to ‘Swallowing Disorders’	Relevant to ‘Behavioral Therapy’	Relevant to the BOT-PPW approximation
Deglutition disorder*	Exercise therapy/program	Oropharyngeal pressure
		Pharyngeal pressure
Swallow*	Rehabilitat*	Base of tongue pressure
		Tongue base pressure
Dysphagia*	Non-surgical therapy	Posterior pharyngeal wall pressure
Bolus driving pressure	Tongue-hold	Base of tongue to posterior pharyngeal contact duration
	Tongue-hold	
	Tongue-hold	
Pharyngeal constrict*	Masako exercise	Approximation
	Masako maneuver	Contact
	Masako maneuver	Duration
Deglutitive failure	Lingual exercise	
	Lingual strengthening	
	Tongue exercise	
	Tongue strengthening	
Base of tongue	Surface EMG activity	
Deglutition	Rehabilitative exercise	
Sarcopenia	Effortful swall*	
Tongue base	Tongue-hold*	
Pharyngeal tongue	Hyolaryngeal muscle activat*	
Posterior pharyngeal wall	Age-related muscle weakness strength training exercise	
Base on tongue to posterior pharyngeal wall contact	Strength training*	
Base of tongue to posterior pharyngeal wall approximation	Treatment	
Tongue base retraction	Muscle stretching exercise	
Posterior pharyngeal wall pressure		
Posterior pharyngeal wall approximation		

An asterisk was used in the literature searches to denote a wildcard search for the respective search term

All publications identified across the six databases were imported into the software platform Covidence for screening [18]. In addition, any relevant references identified from the reference lists of included studies were also screened for inclusion. We initially focused our analysis on studies that reported effects of interventions that were performed repeatedly over a period of time:

- Review Part 1 focused on publications that (i). reported on outcome measures specifically representing BOT-PPW approximation, (ii). reported on a behavioral intervention, and (iii). that behavioral intervention had been implemented over a period of time in a rehabilitative approach (i.e., cumulative, long-term effects).

Due to the very small number of studies identified in this initial screening, we repeated the screening process with a re-focused scope, including studies that reported on the immediate effects of interventions that were performed in a one-off manner and had a ‘proof of concept’ focus:

- Review Part 2 focused on inclusion of publications that (i). reported on outcome measures specifically representing BOT-PPW approximation, (ii). reported on a behavioral intervention, and (iii). the effects of that behavioral intervention were investigated *during* performance of that intervention in a one-off session (i.e., immediate effects).

For the purpose of both Review Parts, we defined behavioral intervention as an exercise that was actively and repeatedly performed *by* the patient (e.g., a maneuver or exercise) as opposed to interventions that are performed *on* the patient, (e.g., surgery). The potential role of surgical, medical, or neuro-stimulation approaches in rehabilitating swallowing function is acknowledged. However, the intent of this scoping review was to collate the evidence base for behavioral interventions that can be completed by patients themselves. Therefore, studies were excluded if they (i) described a surgical or other medical intervention, (ii) did not directly report on objective outcome measures biomechanically representing BOT-PPW approximation (e.g., reported on reduced residue without reporting an objective outcome measure representing BOT-PPW approximation), (iii) involved brain, magnetic, or electric stimulation, or (iv) provided no experimental primary data (e.g., reviews or expert opinions).

Title and abstract screening, as well as full-text screening for both Review Parts were undertaken independently by two researchers, who were blinded to the other reviewer’s recommendation. Any conflicts were resolved by consensus. Studies that met the inclusion criteria proceeded to data extraction for each Review Part.

### Data Extraction

For both Review Parts, data from included studies were extracted and collated in an Excel document by at least two researchers, who resolved any conflicts by consensus. Data relating to study design, participant characteristics (sample size, age, diagnosis), intervention evaluated, and BOT-PPW approximation outcome measures reported on were extracted (Tables 2 and 3). We only extracted outcome measures that specifically related to BOT-PPW approximation, i.e., measures representing peak contract pressure and/or contact pressure duration (on manometry) or extent of movement or contact duration [on videofluoroscopic swallowing study (VFSS)] at this level of the pharynx. Any additional measures or analyses reported in included studies [e.g., hypopharyngeal pressure, upper esophageal sphincter (UES) related metrics] were not extracted.

**Table 2:** Characteristics and key findings of studies evaluating effects of long-term rehabilitative intervention regimens

Authors	Study design	Intervention	Intervention protocol	Assessment tool	outcomes measures at level of BOT-PPW	Population	Age range (mean age)	Key findings	GRADE level of evidence
Juan, [22]	Single case study	I-PRO training	8 weeks of training, 5 weeks of de-training, 9 weeks of maintenance training	HRM	Peak pressure	Dysphagia following multiple embolic strokes (*n* = 1)	59 years	Post I-Pro therapy (week 8) increased peak tongue- pressures and decreased vallecular and PPW residue, but increased cricopharyngeal residue	Very low
Greig, [23]	Single case study	BiSSkiT protocol followed by HRM based biofeedback training	BiSSkiT: 2 weeks, 2 × 1 h per day, 5 days per week; HRM based training (45 min/day, 4–5 days/week for approx. 2 months)	HRM	Peak pressure	Dysphagia following multiple strokes (*n* = 1)	70 years	HRM training increased peak pressures	Very low
Oh, [24]	Randomized controlled trial	TH swallow protocol	4 weeks of TH training, daily for 20 min, every 5 s	VFSS	PCR and PPW anterior movement	Healthy (*n* = 20)	22–33 years (26.4)	PCR and PPW anterior movement unchanged	Moderate

*I-PRO* isometric progressive resistance oropharyngeal, *HRM* high-resolution manometry; *PPW* posterior pharyngeal wall, *BiSSkiT* Biofeedback in Strength and Skill Training, *TH* tongue hold, *VFSS* videofluoroscopic swallowing study, *PCR* pharyngeal constriction ratio

**Table 3:** Characteristics and key findings of studies evaluating immediate effects of swallowing maneuvers

Author	Study design	Intervention	Assessment tool	Outcome measure at level of BOT-PPW	Sample	Age range (mean age)	Key findings	GRADE level of evidence
Intervention 1—effortful swallow								
Pouderoux, [14]	Quasi-experimental	ES v Normal	Strain gauge manometry	Peak pressure	Healthy (*n* = 8)	21–35 years (not stated)	ES increased pharyngeal peak pressure	Low
Lazarus, [25]	Quasi-experimental	ES v Normal	LRVM	Peak pressure on manometry and contact duration on VFSS	HNC (*n* = 3)	65 years, 73 years, 72 years	ES increased pharyngeal peak pressure and increased contact duration on VFSS and nominally decreased upper pharyngeal residue	Low
Hiss, [26]	Quasi-experimental	ES v Normal	LRM	Contact pressure duration	Healthy (*n* = 18)	Not stated (27.9)	ES increased pressure duration	Low
Huckabee, [27]	Quasi-experimental	ES v Normal	LRM	Peak pressure	Healthy (*n* = 22)	Not stated (27.9)	ES increased peak pressure	low
Huckabee, [28]	Quasi-experimental	ES v Normal 1/Tongue emphasis 2/No tongue emphasis	LRM	Peak pressure	Healthy (*n* = 20)	20–35 years (not stated)	ES with “tongue to palate” increased peak pressure more than “no tongue emphasis”	Low
Steele, [29]	Quasi-experimental	ES v Normal 1/Tongue emphasis 2/No tongue emphasis	LRM	Contact pressure duration	Healthy (*n* = 20)	20–35 years (not stated)	Earlier and longer pharyngeal pressure during both ES types indicative of higher velocity bolus driving forces	Low
Witte, [30]	Quasi-experimental	ES v Normal	LRM	Peak pressure	Healthy (*n* = 40)	20–43 years (25.8)	No change in pharyngeal peak pressure and pressure duration during ES	Low
Takasaki, [31]	Quasi-experimental	ES v Normal	HRM	Peak pressure	Healthy (*n* = 18)	23–28 years (not stated)	ES increased pharyngeal peak pressure	Low
Hoffman, [15]	Quasi-experimental	ES v Normal	HRM	Maximum pressure at tongue base, contact pressure duration at tongue base, area integral of pressure at tongue base	Healthy (*n* = 14)	19–25 years (21.2)	No change in pharyngeal pressure, pressure duration and pressure integral	Low
Fritz, [32]	Quasi-experimental	ES v Normal	MRI	Duration of pharyngeal closure (ms) during swallow, AP length (mm) during swallow, transverse length (mm) and area (mm^2^) during swallow	Healthy (*n* = 20)	18–30 years (not stated)	ES increased pharyngeal closure duration. All other metrics unchanged	Low
Lenius, [33]	Quasi-experimental	ES (described as increased lingual force during swallow) v Normal	LRVM	Peak pressure	HNC (*n* = 20) (range = 3–179 months post radion therapy)	41–80 years (62)	Increased lingual effort increased peak pharyngeal pressure	Low
Doeltgen, [34]	Quasi-experimental	ES v Normal	HRM	VCI	Healthy (*n* = 12)	21–48 years (28.6)	VCI increased during ES	Low
Jones, [35]	Quasi-experimental	ES v Normal	HRM	Peak pressure at velopharynx, tongue base, and hypopharynx	Healthy (*n* = 9)	21–69 years (42)	ES increased peak pressure at all three levels	Low
Heslin, [36]	Quasi-experimental	ES v normal	HRM	Peak pressure	mixed etiology dysphagia (*n* = 15)	45–86 years (63)	ES increased upper pharyngeal pressure	Very low
Teplansky, [37]	Quasi-experimental	ES v normal	HRM	Peak pressure variability at velopharynx, tongue base, and hypopharynx	Healthy (*n* = 51)	not stated (31.5)	ES increased variability of peak pressure at all three levels	Low
Intervention 2—Mendelsohn Maneuver								
Lazarus, [25]	Quasi-experimental	MM v Normal	LRVM	Peak pressure on manometry and contact duration on VFSS	HNC (*n* = 3)	65 years, 73 years, 72 years	MM increased pharyngeal peak pressure and increased contact duration on VFSS and nominally decreased upper pharyngeal residue	Low
Hoffman, [15]	Quasi-experimental	MM v Normal	HRM	Peak pressure, pressure integral and pressure duration	Healthy (*n* = 14)	19–25 years (21.2)	MM decreased pharyngeal pressure. Pressure duration and pressure integral were unchanged	Low
Doeltgen, [34]	Quasi-experimental	MM v Normal	HRM	VCI	Healthy (*n* = 12)	21–48 years (28.6)	MM increased VCI	Low
Teplansky, [37]	Quasi-experimental	MM v normal	HRM	Peak pressure variability at velopharynx, tongue base, and hypopharynx	Healthy (*n* = 28)	not stated (22.4)	MM increased variability of peak pressure at all three levels	Low
Intervention 3—tongue hold swallow								
Fijiu, [40]	Quasi-experimental	TH v Normal	VFSS	1. extent of PPW bulging during BOT-PPW contact; 2. duration of BOT-PPW contact. 3. vallecular residue (3 ml barium swallows)	Healthy (*n* = 10)	19–26 years (23)	TH increased anterior PPW bulge but BOT-PPW approximation did not last longer. TH increased vallecular residue	Low
Lazarus, [25]	Quasi-experimental	TH v Normal	LRVM	Peak pressure on manometry and contact duration on VFSS	HNC (*n* = 3)	65 years, 73 years, 72 years	TH increased pharyngeal peak pressure and increased contact duration on VFSS and nominally decreased upper pharyngeal residue	Low
Doeltgen, [41]	Quasi-experimental	TH v Normal	LRM	Peak pressure, pressure duration	Healthy (*n* = 40)	20–45 years (not stated)	TH swallows decreased peak pharyngeal pressure; TH swallows did not change pressure duration	Low
Doeltgen, [42]	Quasi-experimental	TH v Normal	LRM	Peak pressure, pressure duration	Healthy (*n* = 68) Y (*n* = 34) O (*n* = 34)	Y 18–40 years (26.8) O 60–84 years (72.6)	In both age groups, TH decreased peak pharyngeal pressure, but did not change pressure duration	Low
Hammer, [43]	Quasi-experimental	TH v Normal (1. tongue at lips; 2. tongue-hold maneuver)	EMG, HRM	SPC EMG, peak pressure	Healthy (*n* = 8)	20–27 years (not stated)	Both maneuvers increased EMG of SPC before and during the swallow; TH prolonged SPC EMG. Neither maneuver affected peak pharyngeal pressure	Low
Teplansky, [37]	Quasi-experimental	TH v normal	HRM	Peak pressure variability at velopharynx, tongue base, and hypopharynx	Healthy (*n* = 35)	Not stated (34.4)	TH increased variability of peak pressure at all three levels	Low
Intervention 4—super supraglottic maneuver								
Ohmae, [45]	Quasi-experimental	SSG v Normal	VFSS and video-endoscopy	Onset of contact relative to UES opening and contact duration	Healthy (*n* = 8)	20–28 years (not stated)	SSG resulted in later BOT-PPW contact. SSG did not change contact duration	Low
Lazarus, [25]	Quasi-experimental	SSG v Normal	LRVM	Peak pressure on manometry and contact duration on VFSS	HNC (*n* = 3)	65 years, 73 years, 72 years	SSG increased peak pharyngeal pressure and contact duration and nominally decreased upper pharyngeal residue	Low
Intervention 5—tasks imitating BOT to PPW approximation								
Veis, [46]	Quasi-experimental	1. Tongue pull back 2. yawn + tongue pull back 3. Gargle + tongue pull back	VFSS	Distance between BOT and mid or inferior C2	Mixed etiology dysphagia (*n* = 20)	38–89 years (61)	Gargle resulted in the shortest mean distance between BOT and mid C2. All three tasks increased BOT retraction	Low

*BOT-PPW* base of tongue to posterior pharyngeal wall, *ES* effortful swallow, *LRVM* low-resolution videomanometry, *VFSS* videofluoroscopic swallowing study, *HNC* head neck cancer, *LRM* low-resolution manometry, *HRM* high-resolution manometry, *MRI* magnetic resonance imaging, *ms* millisecond, *AP* anterior–posterior, *mm* millimeter, *VCI* velopharyngeal contractile integral, *MM* Mendelsohn maneuver, *TH* tongue hold, *PPW* posterior pharyngeal wall, *Y* young, *O* old, *EMG* electromyography, *SPC* superior pharyngeal constrictor, *SSG* super supraglottic swallow; *UES* upper esophageal sphincter, *BOT* base of tongue, *C2* cervical vertebra 2,

### Presentation of Key Findings

In order to answer the primary research question, we grouped the characteristics of the reported interventions into broader themes that best captured similarities and differences across interventions. This was undertaken in an iterative cycle of refinement through discussion by the research team.

### Assessment of Study Design and Quality

Two assessors independently assessed the risk of bias of each included study using the critical appraisal tools from the Joanna Briggs Institute (JBI) [19], with any discrepancies resolved by group consensus. We then evaluated the strength of each study using the Grading of Recommendations, Assessment, Development and Evaluation (GRADE) framework, which rated evidence quality across four levels: very low, low, moderate, and high [20, 21]. Two team members determined if the recommended GRADE starting point for each study (e.g., “high quality” for randomized controlled trials, “low quality” for cohort or experimental studies) should be up- or down-graded depending on methodological rigor identified by the JBI criteria.

## Results

**Fig. 1:**
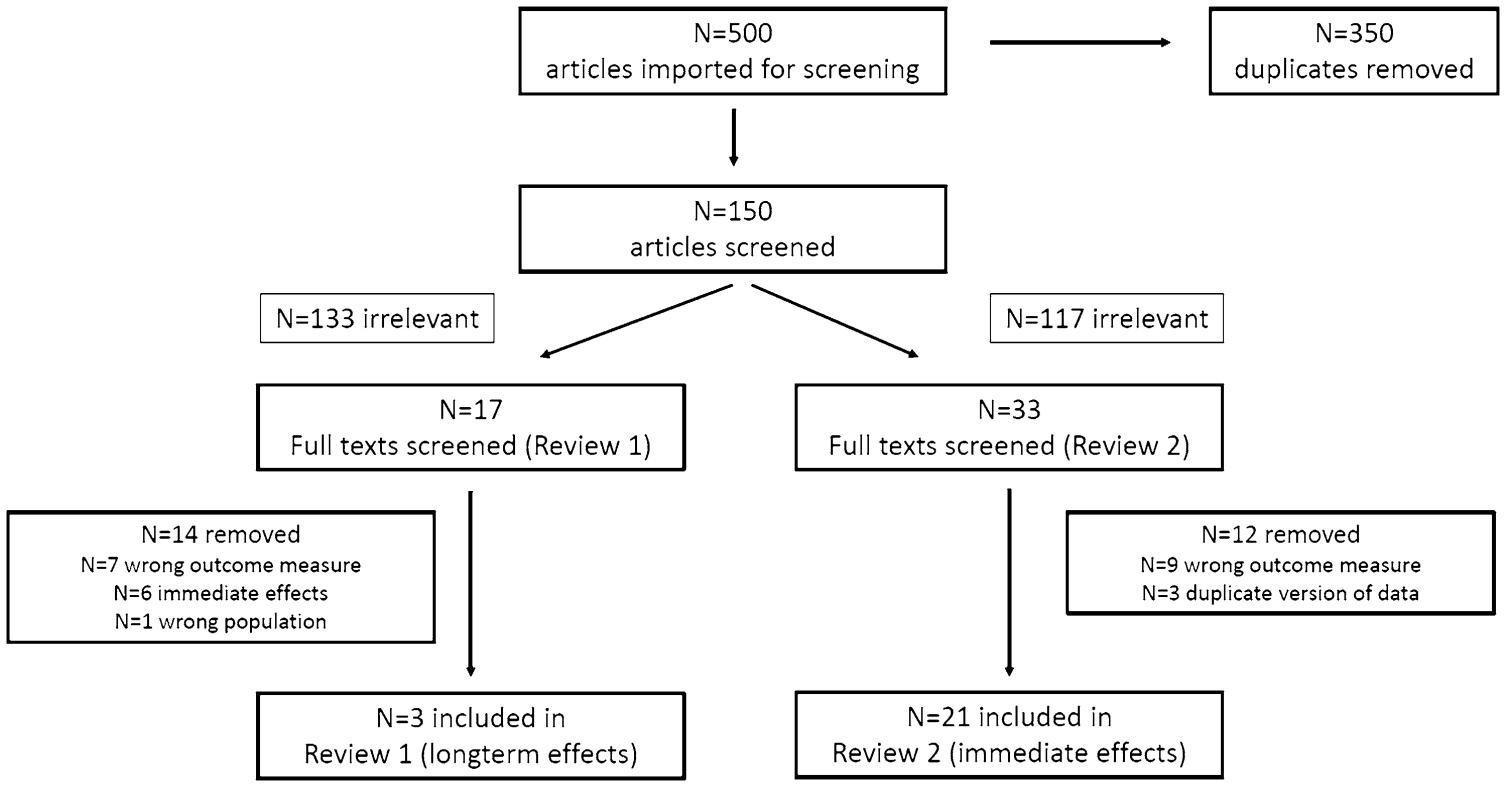
Preferred Reporting Items for Systematic Reviews (PRISMA) diagram illustrating outcomes of the literature search and screening processes

After deduplication, 150 studies underwent title and abstract screening in the Covidence software, of which 17 studies proceeded to full-text screening for Review Part 1 and 33 studies for Review Part 2. Of these, three studies met the inclusion criteria for Review Part 1 and 21 studies for Review Part 2 (Fig. 1).

### Summary and Discussion of Studies Evaluating Effects of Interventions Addressing BOT-PPW Approximation

#### Review Part 1—Long-Term Effects on BOT-PPW Approximation

Across the three included studies, one single case study evaluated the effects of a tongue strengthening paradigm in a patient with dysphagia following multiple embolic strokes [22]. Another single case study evaluated the effects of a biofeedback-assisted swallowing training in a patient with dysphagia following right occipital lobe and posterolateral medullary infarcts [23]. Last, one randomized control trial (RCT) evaluated the cumulative effects of a 20 session, four week long effortful swallowing regimen in healthy volunteers [24]. Both patients with dysphagia demonstrated improved BOT-PPW approximation as reflected in increased contact pressures at the level of the BOT during swallowing [22, 23]. In contrast, the healthy participants in the RCT did not show any changes in the pharyngeal constriction ratio and maximum PPW movement as visually assessed using VFSS following the intervention [24]. Both single case studies represent very low level evidence for the evaluated interventions, whereas the RCT contributed low level evidence as per the adjusted ratings against the GRADE framework.

Table 2 summarizes the findings reported from the three studies included in Review Part 1. Outcome measures that state ‘increased’ or ‘decreased’ denote significant results, where the reported p value was equal or less than 0.05. Those that state ‘unchanged’ reported no significant differences (*p* > 0.05).

#### Review Part 2—Immediate Effects During Execution of Maneuvers

Due to the lack of a strong evidence for the long-term effects of behavioral interventions targeting BOT-PPW approximation, we expanded the scope of this review to include investigations that evaluated the immediate effects of any behavioral interventions on BOT-PPW approximation, providing preliminary proof of concept support for their potential clinical utility. Table 3 summarizes the 21 studies included in Review Part 2.

**Fig. 2:**
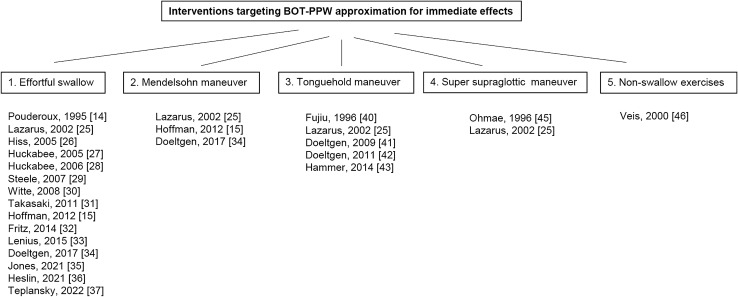
Included studies categorized by intervention type for immediate effects on BOT-PPW approximation

As outlined in Fig. 2, the evaluated interventions were grouped as follows: (1) effortful swallowing, (2) Mendelsohn maneuver, (3) tongue-hold maneuver, (4) super supraglottic swallowing maneuver, and (5) non-swallowing exercises. The following section briefly summarizes and discusses key points pertaining to each of these intervention types, such as the number of studies identified, sample characteristics, and significant BOT-PPW approximation-specific results reported. A broader discussion of implications is provided in the Discussion section.

##### (1) Effortful Swallowing

As a well-established and widely researched swallowing strategy, the effortful swallow is designed to increase global effort during swallowing by means of increased muscle contraction during the pharyngeal phase of swallowing [14]. Fifteen studies evaluated the immediate effects of effortful swallowing on BOT-PPW approximation. Twelve of these 15 studies evaluated healthy participants (total *n* = 299) using pressure-based outcome measures (pressure peak or pressure duration) (*n* = 11 studies) [14, 15, 26–31, 34, 35, 37] or magnetic resonance imaging (*n* = 1 study) [32]. Three studies evaluated immediate effects in patients with dysphagia [25, 33, 36]. Of these, one study investigated the effortful swallow in three patients with HNC [25] using low-resolution videomanometry. All three of these patients presented with BOT-PPW contact pressures that were lower during normal control swallows than what had been reported for healthy controls. The second clinical study evaluated 15 patients with mixed etiology dysphagia using high-resolution manometry (HRM) ([36]. In both of these clinical studies, attempts were made to confirm that patients presented with instrumentally confirmed impairment of BOT-PPW approximation during control swallows either by comparison to published values in healthy controls [25] or comparison to median values within the patient group studied [36]. The third clinical study described an effortful swallowing intervention as involving “increased lingual force” during swallowing [33]. It is likely that this involved a global increase in pharyngeal muscle contraction, as reflected by increased peak pharyngeal pressure during these swallows. Participants in this study included 20 patients with HNC (3–179 months post radiation therapy), who presented with lower peak pharyngeal pressure compared to published values in healthy controls.

As outlined in Table 3, 10 of the 12 studies in healthy participants reported an increase in BOT-PPW approximation, as measured by increased peak pharyngeal contact pressure [14, 27, 28, 31, 34, 35, 37] and/or increased contact duration [26, 29, 32]. Note that of these 10 studies, one study [37] reported increased variability of pharyngeal peak pressures during swallowing. The remaining two of the 12 studies did not find a significant increase in BOT-PPW approximation measures [15, 30]. While the reasons for this are unclear, differences in recording and analysis approaches in these two studies may have contributed.

Overall, the evidence base for the effortful swallow included the largest number of studies in our Review Part 2 of immediate effects on BOT-PPW approximation. However, these should be considered in the context of the overall strength of the evidence base, noting that all 15 studies were rated as “low” or “very low” quality based on the GRADE framework. While robust in their design as within-subject evaluations, they only provide evidence for a compensatory use of effortful swallowing in modifying swallowing biomechanics and mostly in healthy individuals. It is, therefore, impossible to extrapolate these findings to clinical populations with impaired BOT-PPW approximation, for whom the effortful swallow is often recommended clinically as a rehabilitative strategy.

##### (2) Mendelsohn Maneuver

The Mendelsohn maneuver refers to the voluntary prolongation of maximal hyolaryngeal elevation during swallowing [38]. Four studies evaluated this maneuver in the context of BOT-PPW approximation [15, 25, 34, 37]. Three studies evaluated healthy participants (total *n* = 54), of which one reported increased BOT-PPW approximation [34] based on contact pressure recorded using HRM, whereas another reported decreased pharyngeal contact pressure and unchanged pressure duration [15]. The third study reported increased variability of peak pharyngeal pressures during Mendelsohn maneuver swallows compared to normal swallows [37]. The fourth study evaluated three patients with HNC and reported increased peak pharyngeal pressure and increased contact duration on videomanometry [25].

Overall, the evidence base for this maneuver in the context of BOT-PPW approximation is weak and proof of concept studies in healthy participants contradictory. Biomechanically, it stands to reason that prolonged hyolaryngeal elevation during maneuver swallows would require increased and prolonged effort and may, therefore, synergistically modulate other biomechanical aspects of the pharyngeal swallow, including pressure generation at the level of the BOT. However, in the studies included in this review, maneuver swallows did not appear to consistently translate to increased peak pressure and pressure duration at this level, at least in healthy volunteers and when measured during maneuver swallows. Therefore, at present, it is not possible to draw strong conclusions from these findings as to whether the Mendelsohn maneuver may improve impaired BOT-PPW approximation in patients with dysphagia, e.g., if performed repeatedly over a period of time. Future high-quality research is required to evaluate its efficacy in this context.

##### (3) Tongue-Hold Maneuver

Increased PPW contraction may be targeted to compensate for reduced BOT retraction [39]. As such, the original premise of the tongue-hold (or Masako) maneuver is to increase the anterior bulge of the PPW during swallowing by anteriorly anchoring the tongue between the incisors [40]. In Review Part 2, six studies were included that evaluated the tongue-hold maneuver [25, 37, 40–43], of which five studies evaluated healthy participants (total *n* = 161) [37, 40–43]. Of these five studies, two reported decreased peak contact pressure during tongue-hold swallows [41, 42] and unchanged pressure duration. Another study reported unchanged peak pressures in the presence of increased and longer superior pharyngeal constrictor activity based on electromyography [43], which is in line with the original publication on this maneuver reporting increased PPW bulging [40]. The fifth study reported increased variability of peak pharyngeal pressures during tongue-hold swallows compared to normal swallows [37]. In three patients with HNC, tongue-hold swallows increased peak contact pressures and increased contact duration on VFSS [25]).

It is perhaps not surprising that BOT-PPW contact pressures were decreased during tongue-hold swallows in healthy volunteers, as the anterior positioning of the BOT biomechanically limits BOT retraction during swallowing. For this reason, it has been suggested that the tongue-hold exercise should not be used during bolus swallows, as the reduced posterior retraction, and resulting reduced BOT-PPW contact pressure are likely to impede pharyngeal bolus flow [40, 41]. Of note, increased superior pharyngeal constrictor activity [43] and anterior bulge of the PPW [40] may suggest that, at least in principle, this exercise may have positive cumulative effects on BOT-PPW approximation if conducted repeatedly over time. However, to date, no studies in either healthy or dysphagic individuals could be identified that evaluated this premise empirically and further research is needed to evaluate this.

##### (4) Super Supraglottic Maneuver

The super supraglottic swallow maneuver was originally designed to improve laryngeal valving during swallowing, particularly for individuals who had undergone a supraglottic laryngectomy [44]. Individuals hold their breath tightly and bear down while swallowing, and then immediately cough. Two studies, however, investigated the pharyngeal effects of this maneuver [25, 45]. One study evaluated peak pharyngeal pressure and contact duration on videomanometry in three patients with HNC [25]. All three patients presented with reduced BOT-PPW approximation during baseline swallows, and super supraglottic swallows were reported to increase peak contact pressure and pressure duration during this maneuver. The other study evaluated BOT-PPW contact onset timing (relative to UES opening) and contact duration using concurrent VFSS and videoendoscopic evaluations in eight healthy participants [45]. Onset of BOT-PPW contact occurred later during super supraglottic swallows, whereas contact duration was unchanged.

It is worth noting that although this maneuver was originally designed as a compensatory maneuver, it has been postulated that it could also be used as an exercise to improve BOT retraction [44]. To date, any potential rehabilitative benefits of repeated training with this maneuver over a period of time remain to be investigated.

##### (5) Non-swallowing exercises

While 20 of the 21 studies included in Review Part 2 investigated the effects of swallowing-related interventions on BOT-PPW approximation, one study evaluated the effects of maneuvers that target BOT retraction outside of the context of swallowing [46]. These maneuvers included tongue pull back, tongue pull back during a yawn, and tongue pull back during a gargle. Using VFSS, the distance between BOT and mid or inferior C2 was investigated in 20 patients presenting with mixed etiology dysphagia and instrumentally confirmed reduction in BOT-PPW approximation. All three tasks resulted in increased BOT posterior movement compared to normal swallowing. More individuals demonstrated increased BOT retraction during the gargle task, which most often resulted in greater BOT retraction compared to normal swallowing.

Although these maneuvers were performed outside of the context of swallowing, it is worth considering that increased BOT posterior movement during these maneuvers may, if performed repeatedly over time, have a cumulative effect on BOT-PPW approximation. This may translate into improved pharyngeal pressure generation during swallowing, with positive effects on bolus clearance. Of note, it is likely that such potential benefits would primarily be driven by increased contractile strength of the BOT and PPW musculature, and perhaps less so by task-specific neuroplastic reorganization in swallowing-related cortical motor networks. The latter is more likely to contribute to functional changes observed following exercises executed during swallowing tasks, because such exercises address the neuroplastic principle of specificity, which non-swallowing exercises do not [47]. Future research is warranted to further evaluate the effects of these maneuvers when performed in repeated intervention paradigms in patients with dysphagia. In addition, more research is needed to evaluate the neuroplastic changes associated with swallowing (and non-swallowing)-related behavioral interventions in order to better understand the neurophysiological drivers of long-lasting biomechanical improvements.

### Summary of Key Observations

In summary, the following key observations were made in relation to the available evidence base, which may aide the reader in their clinical evaluation and decision making:

(i) Only three studies evaluated the effects of long-term, repeated intervention paradigms, of which two were single case studies of individuals with dysphagia and one was an RCT of 20 healthy controls (Review Part 1).
(ii) In Review Part 2, 21 studies were included, which evaluated the immediate effects of primarily effort-based maneuvers. The majority of these (*n* = 17) studied healthy participants (a total of *n* = 433). Only four studies involved participants with dysphagia (a total of *n* = 58).
(iii) Across both Review Parts, all but one study were within-subject evaluations, mostly of healthy individuals, which provide solid proof of concept evidence for the possibility of behavioral modification of BOT-PPW approximation. However, in terms of the clinical applicability and generalizability of the existing evidence base to long-term clinical improvement, the overall strength of this evidence base in alignment with the GRADE framework is considered weak.

## Discussion

This scoping review evaluated the literature for behavioral interventions that aim to improve BOT-PPW approximation, either via repeated performance of an exercise regimen over time (Review Part 1) or in terms of the immediate effects demonstrated during execution of the maneuver (Review Part 2). This information is important to inform clinical dysphagia management decisions and hence was collated through a systematic search of the evidence base. Overall, this review demonstrates that there is a large body of research suggesting that BOT-PPW approximation can, in principle, be modified using several, in particular effort-based, behavioral maneuvers. However, there is little to no evidence documenting the effects of long-term exercise regimens in patients with dysphagia. In addition to the summary and interpretation of findings outlined above for each intervention type, in the following, we raise several broader issues that sit across both Review Parts and all intervention types for consideration in clinical practice and future research.

### Review Part 1—Long-Term Effects on BOT-PPW Approximation

Only three studies were identified that evaluated the effects of long-term exercise regimens on BOT-PPW approximation, including two single case studies [22, 23] and one RCT in healthy participants [24]. Recent commentary strongly recommends that management of dysphagia extend beyond compensatory approaches to skill and strengthening exercises, in line with exercises for other sensory or motor impairments [48–50]. The lack of evidence regarding the effects of long-term exercise regimens perpetuates non-targeted rehabilitative practice and reliance on compensatory measures.

### Review Part 2—Immediate Effects During Execution of Maneuvers

We identified five types of interventions, which included (1) effortful swallowing, (2) Mendelsohn maneuver, (3) tongue-hold maneuver, (4) super supraglottic swallowing maneuver, and (5) non-swallowing exercises that demonstrated immediate effects during execution of maneuvers. Four of these intervention types are executed in the context of swallowing and aim to increase BOT-PPW approximation primarily via increased peak effort or longer effort duration, or manipulation of anatomical position of the tongue, which in turn stimulates a more effortful contraction of the PPW to compensate. The fifth intervention type is characterized by movements that occur outside the context of swallowing, but similarly aim at increasing muscle strength through posterior movement of the tongue.

It stands to reason that volitionally increasing contractile strength and vigor during swallowing would modify BOT-PPW approximation through synergistic modulation of muscle contraction across the swallowing network that is governed by central pattern generators in the brainstem and modifiable by cortical sensorimotor networks [51]. As such, therapeutic benefit may not only arise from increased muscle strength, but also from neuroplastic reorganization in swallowing-related cortical sensorimotor networks, at least in exercises that are executed in the context of swallowing. For example, effortful and Mendelsohn swallowing have been shown to be associated with significantly greater activation of cortical regions related to swallowing in healthy volunteers [51]. Thus, repeated execution of swallowing-related exercises is likely to induce neuroplastic reorganization in these networks, providing a promising neurophysiological mechanism by which longer term biomechanical improvements may be maintained.

The proof of concept evidence collated in this review largely appears to support the notion that cortically driven modulation of the swallowing motor response may yield beneficial biomechanical effects downstream. It is likely that for many patients with dysphagia who present with instrumentally demonstrated reduction of BOT-PPW approximation, and related pharyngeal bolus residue, effort-based maneuvers may, in principle, increase BOT-PPW approximation and in turn reduce pharyngeal residue. However, as outlined in Review Part 1, there is virtually no empirical evidence to support this hypothesis, and therefore, we propose that this should be an urgent key focus of future clinical research.

We also raise the following point for consideration: it is unlikely that reduced BOT-PPW approximation is always the result of pharyngeal tongue weakness in patients with this biomechanical impairment. It is possible that in some individuals, heightened muscle tone and/or spasticity lead to the reduced dynamic movement of these structures during swallowing. For these patients, interventions focusing on increased effort will likely exacerbate their swallowing impairment, and therefore, diagnostic precision is critical in developing tailored dysphagia management plans.

### Diagnostic Precision

Most studies included in this review utilized quantitative assessment methods, primarily manometric, to evaluate changes in BOT-PPW approximation, using peak pressure or pressure duration as proxy measures for the contact of these structures during swallowing. Using objective, quantitative outcome measures are critical, as secondary functional outcome measures, such as pharyngeal bolus flow, degree of penetration or aspiration, or amount and location of residue are more meaningful in the context of clearly characterized changes in the underlying biomechanical processes. In addition, it is critical that future research evaluates individuals who present with the specific biomechanical swallowing impairment that the exercise is designed to target and that this impairment (in the case of this review, reduced BOT-PPW approximation) be instrumentally confirmed prior to inclusion in the research. While proof of concept studies in healthy controls can demonstrate immediate biomechanical changes, their use in determining longer term effects is limited as in an unimpaired system with optimized BOT-PPW approximation, there is little physiologic need, or scope, for change.

### Objective Assessment of BOT-PPW Approximation

In the majority of included studies, BOT-PPW approximation metrics were assessed using low- or high-resolution pharyngeal manometry, or VFSS (Table 3). Across these studies, various protocols were employed, including different manometry catheter designs (3–36 sensors), bolus volumes (saliva and 3 ml, 5 ml, 10 ml, 20 ml), consistencies (thin, nectar, pudding, thick), bolus types (water, barium, saliva, mashed potato, conductive test jelly), as well as analysis approaches. Given demonstrated differences in pressure dynamics and bolus flow in response to all of these variables, clarity in reporting the exact procedures used and careful interpretation of the findings of this scoping review are critical when considering these data during dysphagia management decisions.

In addition, we propose that future clinical research exploring the effects of long-term treatment regimens may consider using assessment tools that can simultaneously evaluate changes in BOT-PPW approximation and corresponding effects on bolus flow. It is possible that the limitations of quantifying BOT-PPW approximation using videofluoroscopy alone may have contributed to the lack of evidence for this important biomechanical bolus driving force. HRM with impedance or videomanometry can both contribute this information to the evidence base. Intervention studies using HRM with impedance or videomanometry are critical in populations with instrumentally confirmed BOT-PPW approximation impairment, given that previous research has documented the critical role of upper pharyngeal pressure in generating the bolus driving forces that facilitate bolus passage across the UES [10]. Due to its higher spatial resolution, HRM with impedance is likely to provide superior measurement of changes in BOT-PPW biomechanics than low-resolution manometry, which relies on pressure recordings from a single sensor in the target area.

### Clinical Recommendations and Future Directions

Given the small number of clinical intervention studies identified in this review, it is at this stage not possible to form a clear recommendation for or against any of the identified exercises. As outlined in Tables 2 and 3, a total of only 60 patients with dysphagia have to date been evaluated for the effects of behavioral interventions on BOT-PPW approximation, of which 58 patients were evaluated for immediate effects during one-off execution of an intervention. This represents a very small sample size, especially in the context of the clinical heterogeneity in the underlying medical conditions of the included individuals.

The inability to make definitive clinical recommendations on the basis of this review highlights the importance of future clinical intervention research in this space. This is a key requirement to facilitate the translation of the proof of concept research evidence base in healthy participants, which demonstrates promising potential for several intervention approaches, into clinically useful application. The challenges of clinical research in this space are acknowledged and likely relate to the recruitment of participants with dysphagia due to homogenous medical conditions and the availability of instrumental assessments in clinical research contexts. Only two clinical studies which reported biomechanical outcome measures also reported clinical outcomes. We propose that future clinical studies should report both clinical outcome measures such as residue, penetration or aspiration, as well as objective measures that qualitatively characterize biomechanical changes as primary outcomes. Collaboration of multiple clinical sites will likely facilitate future research that uses clearly defined clinical research protocols in patients presenting with dysphagia characterized by impaired BOT-PPW approximation. Clinical trials in patient populations are particularly important as there is little need for physiologic change in unimpaired systems, and translation of any effects found in an unimpaired system to dysphagic populations is limited.

## Limitations

We acknowledge that, although non-traditional, the focus of this review was intentionally twofold, focusing both on long-term and immediate effects of interventions claiming to target BOT-PPW approximation. We limited the inclusion of studies to those directly reporting on primary biomechanical outcome measures that represent BOT-PPW approximation. We excluded any studies that reported on related, secondary outcome measures, e.g., pharyngeal residue or degree of penetration/aspiration, if no primary outcome measures were reported. This may slightly underestimate the existing evidence base but was considered important in the context of answering the research question. Second, only studies published in English were included, possibly resulting in some relevant research published in other languages being overlooked.

## Conclusion

This review identified five broad intervention types addressing BOT-PPW approximation, most of which have been evaluated for their immediate, compensatory effects on pharyngeal swallowing biomechanics in healthy individuals. Overall, the evidence base for these interventions is weak, necessitating further high-quality research to address current limitations. Exploring longer term intervention protocols in populations with demonstrated BOT-PPW approximation impairment will facilitate transfer into clinical application.
